# Indents

**Published:** 1904-01-15

**Authors:** 


### INDENTS.

☞ **Brief Articles of Technical or Scientific Value will receive notice and comment. Contributions invited.**

**Investment Compound.** Plaster of Paris, two parts, and Portland cement, one part, makes the quickest setting and most reliable investment I have ever used.—Sylvester Moyer, in *Review*.

**Contouring Matrices.** Dr. Hodson suggests that matrices may be easily contoured to reproduce the outlines of the teeth by stamping them with a properly-shaped steel punch on a lead block.

**Cotton Points.** Cut the cotton root-canal points made by Johnson & Johnson obliquely from end to end and get two pointed cotton points, which can be used more successfully in roots than the usual rolls.—Dr. Hodson, in *Review*.

**Painful Dentition.**

| | |
|---|---|
| Cocaine Hydrochlorate | 1 grain |
| Comp. tr. opium | 1 ounce. |

Rub a little on gums when child is restless. Use cautiously.—*Merck's Report*.

**An Aid in Carrying Pumice.** When using felt cones or wheels in polishing rubber plates, gold crowns, etc., hold a piece of soap against the wet cone before applying the pumice. The soap on the cone prevents too much heat and will also carry the pumice.—D. E. Sheehan, in *Summary*.

**Cavity Liner.** Dr. Broomell suggests the following as a cavity liner in *Dental Brief*: Equal parts of chloroform and alcohol. To this add an equal volume of hydronapthhol. If the cavity is very sensitive fill it with a paste made of zinc oxid and eugenol. Allow this to remain in the cavity several days. You will find in applying that it will harden under moisture, which makes it especially applicable.

**To Produce a Glossy Surface on Wax.** After having made the wax base plate as smooth as possible in the ordinary way, if a hard, glossy surface is desired, coat the surface with chloroform several times and with a small mouth blowpipe go over the wax carefully. The chloroform will cause the surface of the wax to melt at a lower temperature leaving a smooth, hard surface.—C. J. Hadley, in *Review*.

**Relieving Sensitive Dentin.** In the case of cervical cavities which are always very sensitive, I use a local anesthetic hypodermically exactly as for extracting. Put on dam and dry thoroughly, touch with carbolic acid followed by warm air. Use sharp burs and run engine fast. The adjusting of clamp cannot be felt no matter how far below the gum the cavity extends.

It works splendidly with me and is worth trying on your next case.—I. B. Kenney, in *Summary*.

**Neuralgia Remedies.**

| | | |
|---|---|---|
| 1—Menthol | 45 | grn. |
| Cocaine | 15 | grn. |
| Chloral Hydrate | 10 | grn. |
| Petrolatum | 300 | grn. |
| 2—Tincture Aconite | 5 | parts. |
| Tincture Chloroform | 5 | parts. |
| Lard | 20 | parts. |
| 3—Oil Peppermint | 8 | parts. |
| Tincture Aconite | 4 | parts. |
| Chloroform | 2 | parts. |
| Poison! Apply every half hour. | | |
| 4—Camphor | 1 | part. |
| Cloral Hydrate | 2 | parts. |
| Chloroform | 4 | parts. |
| Alcohol | 4 | parts. |
| Poison! | | |

—*Merck's Report.*

**A Quick Method of Making a Counter Die.** Take the impression with a compound composed of two parts plaster and one part molder's sand. Dry the impression thoroughly and pour in the die metal as cool as possible. When the metal has crystallized remove the die from the impression. Soften some modeling compound and place it in the bottom of a vulcanite flask. Press the die into the modeling compound, cool in cold water and separate. You are now ready to swage the metal plate. By this method lead counter-dies are dispensed with, and as all the appliances are in the office it will cost nothing to try it.—A. S. Waltz, in *Review*.

**Melanatic Cancer of the Face.** Jaboulay (*La Semaine Medicale*) emphasizes the importance of non-interference with moles. The presence of a mole from its contained pigment should always warn a physician of the danger of melanosis. In his paper he mentions two cases in which a mole after being picked and squeezed developed into a malignant growth. One of these patients, a medical student, excised a mole of the hand and died a short time afterward from melanosis. The malignant neoplasm which developed at the site of the mole in his second patient was situated in the region of the right temple and was regarded as inoperable from its extent. He has administered quinine lactate with some temporary benefit.

**Light for Dark Days.** Dr. M. L. Rhein, of New York, has devised a light which gives an abundance of artificial light without the bad influence of a shadow.

The light consists of a cluster of four Holafen globes, pear-shaped, with the bases uppermost, the surfaces of which are so divided that small prisms result in much diffusion of the light. Each globe contains a 32-candle power electric lamp—128-candle power in all. The apparatus is suspended from the wall by means of a bracket and pulleys, with a counter-balance, which permits of its being raised and lowered as desired. Considerable heat is emanated, which is the only objectionable feature.—*Dental Review.*

**Safe Method of Forcing Escharotics Through Fistulous Opening.** Wrap cotton on a smooth broach, saturate with carbolic acid or other escharotic and insert in root-canal. Fill the cavity with gutta-percha, puncture the filling and insert the nozzle of either a water or hypodermic syringe. The medicine can now be forced through by pressure on the syringe, leaving the cotton in the root. In some cases it is advisable to hold the finger on the opening of the fistula until considerable pressure is felt. This expands the whole fistulous tract, allowing the medicine to enter all folds.—J. Mills, in *Review.* It would be a wise precaution to always precede such treatment by forcing a warm, non-irritant, antiseptic solution through the canal.—Ed. REGISTER.

**Removing Pulps.** The following method was demonstrated by Dr. U. D. Billmeyer, before the Chicago Odontographic Society. "Grind to a powder hydrochlorate of cocain crystals on a glass slab with spatula, incorporating enough carbolic acid (95 percent) to make a thick paste. Apply this paste freely over the exposed pulp and inject by placing beeswax (not baseplate wax) in the cavity in such a way as to bring it well over the borders of the cavity, when gentle pressure can be applied on the wax over the paste and exposure until the patient shows signs of sensation. Then discontinue the pressure for a moment until the surface of the pulp becomes slightly deadened, when pressure can be renewed until no sensation is produced, then the pulp is removed with little or no pain."—*Review.*

**Method of Cleansing Root Canals.** When treating a tooth preparatory to filling the root I use a great quantity of water to cleanse the canals. One who has not tried it will be surprised to see how easily and quickly the work is done. With the rubber in position, I place my saliva ejector above the rubber dam instead of below it, and with a Moffatt syringe I wash the cavity of the tooth and the root-canals thoroughly, using formaldehyde in the water, which is removed as fast as it collects in the depression of the rubber dam.

A small platinum point, such as is furnished with the Dunn syringe, attached to the end of the extra tip of the Moffatt syringe, enables one to get pretty well into some root-canals, and be sure that the water goes just where it is intended.—W. A. Johnston, in *Review*.

**Distinguishing the Eucaines.** G. Eigel (*Pharm. Journ.*, No. 3389, p. 482), recommends the following method of distinguishing solutions of the hydro-chlorates of cocaine, *a*-eucaine and *b*-eucaine: 10 Cc. of a 0.1 percent solution of *a*-eucaine hydrochlorate gives a white precipitate with one drop of ammonia; solutions of cocaine hydrochlorate and *b*-cocaine hydrochlorate of the same strength give no precipitate. One drop of a 1 percent solution of *a*-eucaine hydrochlorate with one drop of a 10 percent solution of potassium iodide deposits, in a few minutes, large crystals of *a*-eucaine hydriodide; solutions of cocaine hydrochlorate and *b*-eucaine hydrochlorate of the same strength give no precipitate. One drop of a 1 percent solution of *a*-eucaine hydrochlorate or cocaine hydrochlorate gives a white precipitate with a drop of a 5 percent solution of mercuric chloride, *b*-eucaine gives no precipitate.

